# Health system constraints in integrating mental health services into primary healthcare in rural Uganda: perspectives of primary care providers

**DOI:** 10.1186/s13033-019-0272-0

**Published:** 2019-03-22

**Authors:** Edith K. Wakida, Elialilia S. Okello, Godfrey Z. Rukundo, Dickens Akena, Paul E. Alele, Zohray M. Talib, Celestino Obua

**Affiliations:** 1 Department of Psychiatry, Mbarara University of Science and Technology, P. O. Box 1410, Mbarara, Uganda; 2 Mwanza Intervention Trials Unit, Tanzania National Institute for Medical, Mwanza Centre, Mwanza, Tanzania; 3 Department of Psychiatry, Makerere University College of Health Sciences, Kampala, Uganda; 4 Department of Pharmacology and Therapeutics, Mbarara University of Science and Technology, Mbarara, Uganda; 5 Department of Medical Education, California University of Science and Medicine, San Bernardino, CA USA; 6 Mbarara University of Science and Technology, Mbarara, Uganda; 7 Department of Pharmacology and Therapeutics and Vice Chancellor, Mbarara University of Science and Technology, Mbarara, Uganda

**Keywords:** Primary healthcare, Education system, Human resource, Facilities, Patient flow processes, Health systems

## Abstract

**Background:**

The World Health Organization issued recommendations to guide the process of integrating mental health services into primary healthcare. However, there has been general as well as context specific shortcomings in the implementation of these recommendations. In Uganda, mental health services are intended to be decentralized and integrated into general healthcare, but, the services are still underutilized especially in rural areas.

**Purpose:**

The purpose of this study was to explore the health systems constraints to the integration of mental health services into PHC in Uganda from the perspective of primary health care providers (PHCPs).

**Methods:**

This was a cross sectional qualitative study guided by the Supporting the Use of Research Evidence (SURE) framework. We used a semi-structured interview guide to gain insight into the health systems constraints faced by PHCPs in integrating mental health services into PHC.

**Results:**

Key health systems constraints to integrating mental health services into PHC identified included inadequate practical experience during training, patient flow processes, facilities, human resources, gender related factors and challenges with accessibility of care.

**Conclusion:**

There is need to strengthen the training of healthcare providers as well as improving the health care system that supports health workers. This would include periodic mental healthcare in-service training for PHCPs; the provision of adequate processes for outreach, and receiving, referring and transferring patients with mental health problems; empowering PHCPs at all levels to manage and treat mental health problems and adequately provide the necessary medical supplies; and increase the distribution of health workers across the health facilities to address the issue of high workload and compromised quality of care provided.

**Electronic supplementary material:**

The online version of this article (10.1186/s13033-019-0272-0) contains supplementary material, which is available to authorized users.

## Introduction

The Primary Health Care (PHC) setting is the first point of contact an individual has with the health system [1, 2]. PHC was formally adopted by the World Health Organization (WHO) through the Alma-Ata declaration as the preferred method for providing a comprehensive, universal, equitable and affordable healthcare service, and could reduce stigma, improve access to care, reduce chronicity of mental illness and improve social integration [3–5]. The WHO model of optimal mental health integration proposes that countries build or transform their mental health services to: (i) promote self-care, (ii) build informal community care services, (iii) build community mental health services, (iv) develop mental health services in general hospitals, and (v) limit reliance on psychiatric hospitals [6].

The WHO issued recommendations to guide the process of integrating mental health services into PHC [5] and these have been embraced by various countries [7–10]. However, mental health in primary care has not been realized in most countries especially in the least resourced countries [11–14]. In Uganda, mental health problems contribute 13% of the national disease burden [15–17]. As an effort to adopt the WHO recommendations of integrating mental health into PHC, [5, 13] the Ministry of Health came up with a decentralized structure for the delivery of health services including referral from Health Centre I (HC I, village level), to HC II (Parish level), HC III (Sub-county level), HC IV (County level), through District Hospitals, to Regional Referral Hospitals and to the National Referral Hospital [13, 18].

Health reforms were undertaken to address the mental health burden, including the formulation of the Uganda Minimum Health Care Package (UMHCP), with mental health as a key component to be delivered at all levels of health service delivery [18, 19], and training care providers to identify mental health problems, manage and/or refer complicated cases to higher levels of care [13]. Mental health reforms in Uganda started after the government recognized mental health among the pressing health needs in the country and set up a mental health program to (a) develop policy and guidelines for implementation of mental health services; (b) provide, co-ordinate and educate populations on mental health promotion and prevention; (c) reactivate the mental health coordinating committee at national level and create inter-sectoral committees at district levels; (d) integrate mental health into all the general health services from HC I up to the district hospital (e) establish a system of integrating mental health patients into their families and communities; (f) establish a system to address psycho-social needs arising as a consequence of disaster or conflict situations (g) identify and address effects of violence including violence against women; and (h) introduce a gender responsive mental health component in paramedical, nursing, midwifery and medical school curricula [20].

In an attempt to enhance health system performance in mental healthcare in Uganda, interventions such as Mental Health Gap Action Programme (mhGAP) were piloted in Kamuli, Jinja and Kitgum districts; PRogramme for Improving Mental health carE (PRIME) and the Emerging mental health systems in low- and middle-income countries LMICs’ EMERALD in Kamuli [21, 22]. Ideally, Uganda should be using the WHO mhGAP intervention guide to facilitate the scaling-up of integrated mental health services; however, the integration of mental health services remains challenging in many parts of the country [18, 23]. Anecdotal information from Mbarara Regional Referral Hospital showed frequent unnecessary referrals, and self-referrals of mental health conditions that would have otherwise been treated at the lower health units.

Guided by the Supporting the Use of Research Evidence (SURE) framework [24] (Additional file 1), this study sought to engage to primary healthcare providers (PHCPs) in rural Mbarara district to find out what factors were affecting the integration of mental health services into PHC even as evidence suggests that mental healthcare can be delivered effectively in PHC settings [11, 13, 25, 26].

### Sure framework

The Supporting the Use of Research Evidence (SURE) framework [24] was developed for implementing health changes within Africa. The framework is categorized at five levels: (i) recipients of care, (ii) providers of care, (iii) other stakeholders (including other healthcare providers, community health committees, community leaders, programme managers, donors, policy makers and opinion leaders), (iv) health system constraints, and (v) social and political constraints. This study looked at health systems constraints and thus concentrated on the following domains: (1) accessibility of care, (2) financial resources, (3) human resources, (4) educational system, (5) clinical supervision, (6) internal communication, (7) external communication, (8) allocation of authority, (9) accountability, (10) management and or leadership, (11) information systems, (12) facilities, (13) patient flow processes, (14) procurement and distribution systems, (15) incentives, (16) bureaucracy, and (17) relationship with norms and standards. Results in this manuscript focus on only the domains that were frequently reported on (domains 1, 3, 4, 12, and 13) by the study participants. The SURE framework was used in this study because it has been validated in Africa with regards to the identification of and addressing barriers to implementation of policy options.

## Methods

### Research design

This was a cross-sectional qualitative study that examined the health systems constraints to the integration of mental health services into PHC by PHCPs in rural Mbarara district, Uganda. We used a semi-structured interview guide with probing and open-ended questions that then allowed the respondents to bring in unique examples and detailed descriptions. The interview guide was developed based on the SURE framework [24] using the domains to provide a systematic approach to the identification of barriers.

This approach is methodologically similar but contextually different from our previous publication on barriers to integration of mental health services into PHC in rural Mbarara district of Uganda [27]. The study was designed by EW in consultation with CO, ESO, and ZT.

### Study setting and context

The study was conducted in Mbarara district approximately 270 km (170 miles), southwest of the capital city, Kampala, by road. Mbarara is the administrative capital of southwestern Uganda and it boarders Ibanda and Kiruhura Districts to the north, Kiruhura and Isingiro Districts to the east, Isingiro and Ntungamo Districts to the south, and Sheema District to the west [28].

Mental health services in Uganda are provided at health center (HC) III (sub-county level), with subsequent referrals to HC IV (county level), district hospitals, regional referral hospitals and national referral hospitals [29, 30]. Each health facility level (except HC III) is expected to have general doctors (medical officers), clinical officers (Diploma level Medical Assistants), nurses and midwives, and psychiatric nurses. The HC IIIs do not have general doctors but have all the other cadres of service providers. Mbarara district has thirteen HC IIIs and four HC IV and provision of health services is spearheaded by the district health department responsible for curative and preventive healthcare [31]. All the HCs that were included in this study are located in rural Mbarara district.

### Research participants

Participants in the study included clinical officers, nurses and midwives from six health centers (III and IV); doctors were not part of the study because the HCs either did not have general doctors in the establishment (all HC III), they did not provide consent to participate, or doctors were not available at the time of the study (on official assignment off station). There are four HC IV in Mbarara district and each had only one Medical doctor at the time of the study.

The sampling frame for this study was seventeen HCs (thirteen HC III and four HC IV). Each category (HC III and HC IV) was evaluated for inclusion based on whether they were a government facility, had PHCPs’ who directly assessed patients, and not neighboring a similar health facility (including privately owned), or not located near a hospital. The facilities were randomized by strata (HC III and HC IV) to obtain an equal number of HCs per cluster.

While we proposed to consider age, gender, occupation and seniority/experience when selecting participants, we found on the ground that the health facilities had a smaller health workforce than we had anticipated (about five to eight per HC), thus we recruited all PHCPs we found at the facilities and only interviewed those who provided signed consent. A total of 20 in-depth interviews were conducted, twelve participants were from HC IV and eight from HC III. By the time we interviewed the last participant, there was no new information being generated. We had more females (n = 18) than males (n = 2), and there were no significant age differences between the study participants with ages ranging between 30 and 49 years at the time of the study. In terms of position or health cadre’s levels, we found ten nurses, four midwives, two psychiatric nurses, and four Clinical Officers in the health facilities. Views of all the participants were included in the analysis and they contribute to the conclusions in our study.

### Data collection and analysis

A semi-structured interview guide (Additional file 2) was used to collect data from PHCPs at six HCs. The interviews were conducted in English and the national official language, and backed by field notes. The interview guide was pilot tested at a HC that was not included in the study. The pilot was intended to assess the clarity of the questions, and those that seemed unclear were revised. In-depth interviews were conducted by the lead author (EW) and two trained research assistants (MN and CK) between November 2017 and April 2018. All interviews were conducted in person at the respective HCs, and all participants provided written consent. Each interview lasted approximately 60 min, was audio recorded, and transcribed verbatim by the research assistants. The transcripts were checked by EW against the audio recordings for correctness of information and securely stored; they can only be accessed by EW, ESO and CO.

Data were thematically analyzed [32, 33] by ESO and EW with the help of a qualitative software Atlas.ti version 7 [34]. A codebook was developed by ESO based on the health systems constructs in the SURE framework and the initial coding done by EW after discussion with CO a senior researcher, ZT a health policy expert, AD and PA doctoral team members and GZR a mental health specialist. To address reflexivity, the first author (EW) conducted the initial interviews together with trained research assistants to ensure consistency in the conduct of the interviews. The rest of the interviews were conducted by the trained research assistants (MN and CK). EW conducted the initial analysis of the data, shared and discussed the emerging themes with the rest of the authors.

## Results

A total of 20 in-depth interviews were conducted from six sites in rural Mbarara district with twelve participants from HC IV and eight from HC III. Data presented includes description of the study participants (Table 1), the SURE framework that guided the study (Additional file 1) and the findings from the in-depth interviews. Although the SURE framework presents seventeen domains contributing to the health system constraints, this report focuses on only health systems constraints which featured the most, and they include; accessibility of care (19 times), educational system (18 times), patient flow processes (16 times), facilities (14 times), human resources (11 times), and gender related factors (12 times) as reported by PHCPs in Mbarara district.

**Table 1:** Summary of participants characteristics

(22)	Age	Gender	Health care	Level of education
P1	38	Female	Nursing officer	Diploma in Nursing and Midwifery
P2	41	Male	Clinical officer	Diploma in Clinical Medicine & Degree in Public Health
P3	31	Female	Enrolled nurse	Certificate in Nursing
P4	32	Female	Psychiatric nurse	Certificate in Mental Health Nursing
P5	32	Female	Nursing officer	Diploma in Nursing
P6	45	Female	Nursing officer	Diploma in Nursing & Health service Management
P7	39	Female	Midwife	Certificate in Midwifery
P8	49	Female	Enrolled nurse	Certificate in Nursing
P9	32	Male	Clinical officer	Diploma in Clinical Medicine
P10	30	Female	Midwife	Certificate in Midwifery
P11	35	Female	Enrolled nurse	Certificate in Nursing
P12	49	Female	Nursing officer	Diploma in Nursing and Midwifery
P13	31	Female	Psychiatric nurse	Diploma in Mental Health Nursing
P14	38	Female	Senior Nursing officer	Diploma in Nursing
P15	32	Female	Midwife	Certificate in Midwifery
P16	32	Female	Clinical officer	Degree in Public Health
P17	38	Female	Clinical officer	Diploma in Clinical Medicine
P18	30	Female	Nursing officer	Diploma in Nursing
P19	47	Female	Midwife	Certificate in Midwifery
P20	30	Female	Enrolled nurse	Certificate in Nursing

The majority of the sites (four out of six), where the study was conducted, were led by non-physician providers (three clinical officers and one nursing officer). Two sites had physicians (Medical Officers) who doubled as the facility leaders, but were not available for the interviews. We had anticipated interviewing at least four participants per HC, however, at the time of the study, two out of three HC III had only two individuals meeting the inclusion criteria. Most of the participants were female nurses, aged between 30 and 49 years at the time of the study (Table 1).

We observed that the gender distribution across the HCs visited in Mbarara was unevenly distributed with more females than males.

The other ‘providers of care’ were nursing assistants, health assistants, laboratory technicians and assistants, as well as dispensers. All the sites had both out-patient and in-patients facilities although there were deficiencies in the capacity of beds, safe space for people with mental health problems, and relevant medical supplies as discussed below.

Although our participants across the HCs comprised of fourteen nurses with different levels of training and seniority (2 Senior Nursing Officers and 4 Nursing Officers, 4 Enrolled Nurses, 2 Psychiatric Nurses), 4 Midwives, and 4 Clinical Officers; the results presented in this section generally cut across the participants regardless of health cadre and facility level (HC III or IV). This could be explained by the fact that nearly all PHCPs at different levels performed the same tasks of assessing patients and multitasking in addition to their other roles.

### Accessibility of care

In this study, we looked at accessibility of care in terms of distance to the health facility, financial access and social factors that influence access. One participant mentioned that the hilly terrain prevented clients from accessing care, as reported below:

“…some (clients) are from far but you can see the way this place is, people have difficulty climbing the hills to come. Like the other place behind that hill, it looks near but they have to climb over to come down. The health center may be near the road but it is difficult to access it …whether from near or far, they (clients) have difficulties in moving” **(Nursing Officer, HC IV)**.

The participants noted that their clients faced financial challenges which limited their access to care, especially when they were referred to higher level facilities for specialized management, or when medicines were lacking in the HCs where primary care was being sought. Such clients were reported to be lost to care as they opted to go back home as expressed in the following quote:

“People are poor, they do not have money, so when they come here and you say, that condition I cannot work on it, and you tell them to go to Mbarara (Regional Referral Hospital) or another higher level health centre (HC IV), they will say we do not have money…others may agree (to the referral) but they do not go there, they go back home**…**also when they find that there are no drugs, they cannot come back (to the health centre) they stay at home, then they have their belief that the health workers are not there so they stay there (home)” **(Enrolled Nurse, HC IV)**.

Looking at social factors affecting accessibility of care, participants highlighted poverty and limited support from home as the major issues. That it was not always possible for the clients with mental health problems to go to the HCs for monthly reviews and psychotropic medical refills because the families did not have enough resources to regularly support them. To make it worse, that the families did not feel confident sending the patients unaccompanied because they doubted their ability to reach the health facility alone, and yet financially it was not possible to support both the patients and caretakers. As a result, the caretakers went to pick the refill medications on behalf of the patients. The participants reported this as a challenge because they did not have the opportunity to review the progress of their patients.

“…it (financial situation) is not easy because most of them (clients) are poor…the attendants come without the patient for treatment to pick their monthly refills, which is not good because sometimes we want them to come with patients for review but because of transport problem, they come alone” **(Nursing Officer, HC IV)**.

At a different site however, some participants did not think it was only poverty to blame for the limited access of the clients with mental health problems to healthcare. They noted that there was poor support from home and that the families were not willing to spend money on the people with mental health problems as explained below:

“Of course there is poor support from home; the date for the patient’s review may reach and the people at home tell him that we got no money for you. So this person ends up not coming for medication in time, missing because of no support from home” **(Psychiatric Nurse, HC III)**.

In addition, participants reported limited social support for mental health from the general community including local leaders, citing stigmatization and yet some of the mental health problems are caused by factors within the communities. One respondent attributed it to limited understanding about mental health problems as expressed in the quote below:

“…there is no involvement of political leaders especially when they are in their communities, the counselors, they do not talk about mental health. And even the community involvement, some of the mental illnesses are caused by the community people. The way how they treat their family, the way how they treat community members, it can lead those people to develop mental illnesses. And even the community, when there is a mental patient, they tend to chase that patient away as if that person is mad, they do not look into why that person is behaving like that. But if they have knowledge, they may handle that person, they bring the person to the facility and then after they also give the right care not to induce violence and the patient keeps on getting episodes of mental illnesses” **(Nursing Officer, HC III)**.

### Educational system

Participants across all the sites indicated that they did not receive adequate practical training in mental health as health professional students, and that most of the information was theory based, they were largely cramming to pass exams. As a result of this, the PHCPs expressed a knowledge gap in applying mental health education they learnt into practice. Most of the PHCPs who took part in the study indicated that they had not received any refresher training in mental health since they started practice as illustrated:

“The main challenge is, we lack knowledge…you find like…if I had some mental (health) knowledge in school, which was….you find was also not comprehensive because they would teach say drugs I have never heard about, so you just cram for exams. Now that am in service, I have not had any training about mental health maybe there could be even new interventions but we do not know about them and by the fact that it is not, like no one takes it serious, like the way they train us for HIV, that maybe this is new, this has come like that…so that is the main problem” **(Enrolled Nurse, HC IV)**.

Due to the knowledge deficiencies, most of the participants indicated that they did not feel confident to handle mental health problems. They imagined that only PHCPs specifically trained in mental healthcare provision were supposed to handle clients with mental health problems. However, because of the mental health integration policy and the fact that they are supposed to attend to every client who seeks healthcare, the participants noted that they were left with the option of management by trial and error as expressed in the quote below:

“We don’t have qualified personnel responsible to handle psychiatry. So the facility has no adequate knowledge on handling psychiatric cases. If a new client comes, apart from those who come with a prescribed drug, it is not easy to conclude that this patient needs this or this. It becomes very hard for us, we gamble around, we try a drug, if it works fine…the patient continues with the drug, but if we had someone who understands these mental problems and has experience in handling them, then the patient would be helped” **(Clinical Officer, HC III)**.

### Patient flow processes

We looked at patient flow processes in terms of outreach to the communities and receiving patients, as well as referring and transferring patients. The participants reported that there was neither a structured mental health outreach program at the HCs where the PHCPs go out to the communities, nor a specific way of receiving patients with mental health problems from the communities. All clients were received in the same place in spite of the health condition and followed the same processes. The PHCPs indicated that they did not consider mental health outreach programs as part of their activities, nor was it planned for in terms of finances; instead they waited for the clients to come to them as was expressed by one of the participants:

“We don’t have outreach for mental health…maybe it is because of planning but it is not part of what we do, it needs finances, you cannot go from house to house for free when not catered for in the plan, so outreach is not done” **(Midwife, HC III)**.

The participants however noted that there are Village Health Teams (VHTs) who were supporting them in identifying and referring patients with signs of mental health problems to the HC.

“We have some VHTs that are trained to refer those clients to us, they are sensitized on what mental illnesses are, what the common signs are, so when they find a patient, they refer them” **(Nursing Officer, HC IV)**.

Some PHCPs reported that they were providing talks on epilepsy because it was a very common occurrence in the communities, and that they had educational materials from which the clients obtained information.

“we have some health education talks like that one of epilepsy sometimes we talk about it, then we have posters for example that one of drug abuse, they get information from this side, we don’t go to them” **(Senior Nursing Officer, HC IV)**.

Concerning referral and transfer of patients, the PHCPs indicated that when they were unable to manage a client with mental health problems, they referred them to Mbarara Regional Referral Hospital (MRRH).

“…for an external referral (MRRH), I record in the outpatient register and assign a number; there is provision for where the patient is referred. I then write a referral note for the patient indicating where I am sending them, and a copy is kept here” **(Nursing Officer, HC III)**.

The PHCPs however noted that they were not privy to whether the patients reached the referral place or not because there were no follow-up mechanisms or communication channels with different levels of the health system. They were only able to get back in touch with the clients if they were referred back to the HC for psychotropic medicines refills as illustrated:

“…there is no communication with other care providers after we refer a patient like to Mbarara Regional Referral Hospital. Whether a patient goes, or not, it is up to them. I don’t even get any feedback from where I have referred the patient, except if referred back here for refills…when we refer patients with mental conditions, others go, and others don’t because they have no transport. Those who go and come back are the ones coming for (medicine) refills. Others get medicine once and don’t come back thinking the problem is solved” **(Clinical Officer, HC IV)**.

### Facilities

In this study facilities referred to supply and distribution of necessary supplies and equipment to HCs, and maintenance of these facilities [24]. Participants indicted that there was an existing structure through which medical supplies were provided to the HC and that there was a pre-determined drug list that is followed when supplying medicines to the respective healthcare levels. The participants acknowledged receiving the medicines but deemed them ‘*irrelevant*’ and were shelved in anticipation of what they believed to be relevant for the prevailing mental health problems. In the interim, the PHCPs either referred the clients to where they thought the specific medications were, or asked them to buy.

“…we do not procure drugs they come from NMS (National Medical Stores), it is a push system where they give you what they think you need…we just receive and put in our stores then we wait for another cycle and see whether what we needed previously is there, then we use that. I think the Ministry of Health designed a menu for different facilities so for some drugs you cannot say give me this and they give it to you even if we see that a patient really needs it. The best thing we do is to refer where we think the drug is available and if out of stock, the patient will buy” **(Clinical Officer HC III)**.

In order to address the challenge of medical supplies, PHCPs at HCs which had trained in mental healthcare providers indicated that they had weekly clinic days and that the patients had formed groups where they met whenever they came for review. That in the meetings the patients made regular financial contributions towards buying their psychotropic medicines. The PHCPs reported being supportive to the groups especially in terms of assisting with correct distribution of the medicines, and letting the patient groups keep their medicines and bring them during the next visit to avoid misallocation of the medicines to other patients.

“…most of the drugs have never come however much you order because NMS (National Medical Stores) doesn’t supply them so the patients keep on buying. Patients here contribute money, each month…they have their chairperson, who goes and buys the drugs and then when they come for the clinic, they distribute and even the little we have, maybe other types of drugs we give them also. But like carbamazepine, they (National Medical Stores) don’t usually supply. So even if they supply, it is very little which cannot be enough to meet the need of the clinic…when the patients buy their medicines, we sit with them, and prescribe and then we count the remaining drugs with them and let them keep their drugs because we do not want them to mix with ours, and at another clinic, they come with their drugs” **(Senior Nursing Officer HC IV)**.

Based on the ‘*push system*’ where the NMS has a predetermined drug supply list for medicines per HC level, and the medicines are supplied to the HCs without orders from the HCs. Participants reported that some HCs had medical supplies not available at other facility levels, and could easily share available resources; however, because of no established communication channels between the different levels of the health system, it was not possible to share information. They also noted that the medical supplies had short expiry dates and were not utilized, but because of low motivation, the PHCPs were not willing to share information about the available supplies to avoid expiring on the shelves.

“…they (NMS) give expiring medicines, and also the coordination is difficult to know that the other facility has many drugs. That information is not shared anywhere even though I knew that they were there. What would motivate me to go to the other health unit and pick them I do not see that happening” **(Nursing Officer HC III)**.

Participants who had established clinic days for mental health service provision complained about not having a register to record patient information related to mental health problems as expressed in the quote below. They noted that the general registers did not provide for mental health information thus making it difficult to either integrate mental health services into routine care or providing information to feed into the Health Management Information System (HMIS).

“…we have a challenge of a register (where medical information is recorded), we don’t have a special register for mental health and of which they cannot give me the big red register-the one like for general patients, they used to give me the small books before we had got the bigger ones, but the records person told me they are finished” **(Psychiatric Nurse, HC IV)**.

Participants at one facility reported that the routine transfers of the PHCPs to work in different health facilities, affected provision of service particularly if the only specialized provider is transferred. In this study we found that the only trained mental healthcare provider in one of the facilities was transferred without a replacement. The PHCPs indicated that they were relying on that one individual to provide support for mental healthcare provision; unfortunately, a vacuum was created with the transfer of the staff thus exposing the knowledge gap in the mental health area as evidenced in the quote below.

“…there is some time back when we had a mental health nurse but was transferred. That one was trained purposely for mental people but for me; I don’t have enough knowledge about mental conditions and how to handle those people” **(Midwife, HC III)**.

### Human resources

The participants reported that they were few in number and yet had long queues of clients to attend to in a short time as reported in the quote below. This was evident as the research team had to wait for hours before conducting the interviews. We observed the long queues of patients waiting to be attended to by a handful of PHCPs who were trying their best to attend to each person in the available time.

“…very little time because the clinics are very busy, we have very many patients, who must be assessed for everything, mental and whatever…but sometimes we are not taking full history to identify whether there is mental illnesses so some of the patients are not identified. They remain with their mental problems because of our work load and few staff” **(Nursing Officer, HC IV)**.

The participants expressed concern about the quality of care they were giving to the patients because of the big numbers; some acknowledged low comprehension of the health problem because of mental exhaustion and that they needed specialized mental healthcare providers to support them:

“…Ideally one needs a lot of time to interact with the patient, but there is under staffing and high workload; when we have long queues, the comprehension and right care to every patient is compromised because we are trying to touch here and there to make sure that everyone is satisfied and served in a timely way. There is need for someone who is more trained in mental healthcare and can assess so that we who do the basic refer to that person to attend to the mental health component whenever there is a challenge” (**Clinical Officer, HC III)**.

### Gender related factors

When asked about gender related challenges that would stop the PHCPs from providing mental health services, female participants indicated that they felt unsafe dealing with male clients having mental health problems. They indicated concerns such as fear of aggressive clients which would lead to unintended consequences thus rushing through the assessment; the need for privacy of the male clients (expressed by the female gender); no male psychiatric nurses to provide professional support to the female PHCPs; and being rejected by the male client in preference to a male counterpart. However, there was an appreciation from the participants about the presence of ‘expert clients’, that it gave some female PHCPs confidence about their safety. The ‘expert clients’ provided support such as escorting unaccompanied patients for assessment, handling aggressive patients, and dispensing medicines on behalf of the PHCPs.

“…I think I should be having a male personnel also for mental health concerns because at times you face a challenge, you know these patients need privacy…since I am the only psychiatric nurse here and this person has a psychiatric condition, they will call me to either examine or talk to the patient and of which at times they are naked; I need to have a male person (PCP) to be on a safer side. Anything can happen within the screen…or this person will say, “I don’t need you as a female nurse, I need a male nurse” but he is not there in provision of psychiatry” **(Psychiatric Nurse, HC IV)**.

## Discussion

In this study, we focused on health systems constraints to the integration of mental health services into PHC. It is through health systems that the policy option either flourishes or fails. We were particularly interested in the perceptions of ‘providers of care’ who are expected to implement the policy option. The first noteworthy observation was that in an integrated system, all PHCPs are expected to recognize persons with mental health problems as part of history taking and manage them accordingly. Meaning even the psychiatric nurses and psychiatric clinical officers at the health facilities are expected to offer mental health services alongside other services. This was not the case in health facilities where mental health providers were integrated in the PHC workforce; their presence was misunderstood by the other PHCPs who became reluctant to taking on mental healthcare in their practice. Secondly, the mental health providers at the PHC facilities considered deployment to perform immunization, and family planning programs as outside their area of specialized training. This was a demotivating factor to the mental health providers at the health facilities.

In reference to accessibility to care and the fact that there is limited support at community and family levels as well as stigma, psycho-education interventions targeting the family and community may be helpful in facilitating the much needed integration of mental health services in PHC.

Methodologically, we used the SURE framework [24] to provide the structure and analysis of the study. The framework was developed to support strategies for improving access to and use of research evidence in health policy development in Africa [35]. Findings from this study therefore have potential of contributing to well-informed policy decisions not only in Uganda but African health systems. We did not find a study that used a similar approach to identify health systems constraints particularly faced by PCP. So we believe this was an innovation in this study.

Results from our study highlight accessibility of care, educational system, patient flow processes, facilities, human resources, and gender related factors as important health systems constraints to integration of mental health services into PHC from the perspectives of PHCPs in rural Mbarara district. Although all these factors are in conformity with what other studies have found, contextualization is very important in order to address them. Poor or low accessibility of care for patients with mental health problems may have been cited by various researchers, [7, 36–43] however, the contexts within which these constraints occur are unique to communities and should not be looked at in isolation. In this study, low financial resources, difficult geographical terrain, and family indifferences towards mental illness compounded and contextualized the challenge of low accessibility of mental healthcare services in rural Mbarara district. Similarly, inadequate patient flow process as identified in this study and others [37, 39, 40, 44–51] contextually contributed to the poor accessibility of care. When we looked at these findings in relation to the SURE framework, we noted that adequate processes for outreach and receiving, referring and transferring patients is needed to address this constraint in Mbarara district.

In order for the PHCPs to effectively integrate mental health services into the care of patients at the PHCs, there is need for knowledge and skills in the management of mental health problems. Our findings showed that there remains knowledge and skills gaps as is evidenced in literature from other studies [36, 37, 39, 40, 49–54]. Continuous medical education related to mental healthcare and management will go a long way in addressing these gaps as suggested by the PHCPs who decried lack of in-service training to update their knowledge and skills.

Concerning facilities, although there was an established system through which medical supplies were delivered to the various HCs in Uganda, the PHCPs were disgruntled about it. They felt that their opinions were overlooked and yet they are the implementers on ground who knew the needs of the users (patients). Better communication systems between the HCs (providers of care) and the distributors are therefore called for in order to expand access to mental healthcare services including relevant medical supplies.

The human resource related issues identified in this study were comparable to findings in a systematic review conducted by Wakida, Talib [36] which highlighted low interest in delivering mental healthcare [39, 52] such as of heavy workload and limited time [37, 39, 46, 47, 49, 52–54]. This is a difficult constraint to handle with the low recruitment in Uganda due to limited financing of the health sector at 9.6% compared to the 15% government of Uganda committed to at the Abuja declaration [55].

On the gender front, we found that although there was fear among female participants when dealing with male clients who had mental health problems, they drew more confidence in the presence of ‘expert clients’. The presence of these ‘expert clients’ additionally worked to support the overburdened PHCPs as they fulfilled functions that would have otherwise have been difficult fulfill, and at no cost to the HCs. This scenario is comparable to what happens in HIV/AIDS clinics where expert clients help overburdened healthcare providers to manage the well-attended ART programs without any pay, but provided acquired preferential status because of their knowledge of AIDS medicines and the compassionate care they provide [56]. The distribution of a cross section of the health cadres would also help reduce the burden on the PHCPs thereby improve the integration of the mental healthcare services into PHC in rural Mbarara District and possibly beyond.

## Limitations

The results presented in our study are views from only clinical officers, nurses and midwives; we do not have the views of medial officers because they either did not consent to take part in the study or were not available at the time of the study. Furthermore, since nurses and clinical officers are the frontline practitioners at the lower health facility levels, we felt they were the most appropriate cadre to sample.

Given that this was a case study of one district in one region of Uganda, we cannot entirely generalize the findings to other settings. There may be need for similar studies to be carried out in the other regions of the country to confirm our findings.

## Conclusion

In order to achieve effective integration of mental health services into PHC in rural Mbarara/Uganda, there is need for some health system changes in the following areas: (i) modification of the education system for the health workers to make the training in mental healthcare more comprehensive and practical, and to introduce regular in-service training in mental healthcare delivery for the PHCPs; (ii) institute adequate processes for outreach and receiving, referring and transferring patients with mental health problems—allowing for internal communication between different levels of the health system as well as the community; (iii) revisit the restrictions and empower PHCPs at all levels to manage and treat at least all mental health problems and only refer when it is inevitable (although a supervisory body may be needed). This may in the long run facilitate a smooth mental health integration process; (iv) adequately provide the necessary medical supplies based on the care provider requests; and (v) increase the distribution of health workers across the Health Centre’s to address the issue of high workload and compromised quality of care provided.

## Additional files

**Additional file 1.** Description of the SURE Framework.

**Additional file 2.** Interview guide.

## Abbreviations

DHO: District Health Officer
GUREC: Gulu University Research Ethics Committee
HC: Health Center
PHCP: primary care provider
PHC: primary health care
Sida: Swedish International Development Cooperation Agency
SURE: Supporting the Use of Research Evidence
UMHCP: Uganda Minimum Health Care Package
UNCST: Uganda National Council for Science and Technology
WHO: World Health Organization
