# Supplementary material for: Health system constraints in integrating mental health services into primary healthcare in rural Uganda: perspectives of primary care providers
Source: Int J Ment Health Syst. 2019 Mar 22;13:16. doi: 10.1186/s13033-019-0272-0 (PMC6429816; doi:10.1186/s13033-019-0272-0)
Supplement: Supplementary file 1 — Additional file 1. Description of the SURE Framework. [file 13033_2019_272_MOESM1_ESM.docx]

# Additional File 1: Description of the SURE Framework

| **Level** | **Factors affecting implementation** |
| --- | --- |
| Recipients of care | Knowledge and skills |
|  | Attitudes regarding programme acceptability, appropriateness and credibility |
|  | Motivation to change or adopt new behaviour |
| Providers of care | Knowledge and skills |
|  | Attitudes regarding programme acceptability, appropriateness and credibility |
|  | Motivation to change or adopt new behaviour |
| Other stakeholders (including other healthcare providers, community health committees, community leaders, programme managers, donors, policy makers and opinion leaders) | Knowledge and skills |
|  | Attitudes regarding programme acceptability, appropriateness and credibility |
|  | Motivation to change or adopt new behaviour |
| Health system constraints | Accessibility of care |
|  | Financial |
|  | Human resources |
|  | Educational system |
|  | Clinical supervision |
|  | Internal communication |
|  | External communication |
|  | Allocation of authority |
|  | Accountability |
|  | Management and or leadership |
|  | Information systems |
|  | Facilities |
|  | Patient flow processes |
|  | Procurement and distribution systems |
|  | Incentives |
|  | Bureaucracy |
|  | Relationship with norms and standards |
| Social and political constraints | Ideology |
|  | Short-term thinking |
|  | Contracts |
|  | Legislation or regulations |
|  | Donor policies |
|  | Influential people |
|  | Corruption |
|  | Political stability |
