# Supplementary material for: Health system constraints in integrating mental health services into primary healthcare in rural Uganda: perspectives of primary care providers
Source: Int J Ment Health Syst. 2019 Mar 22;13:16. doi: 10.1186/s13033-019-0272-0 (PMC6429816; doi:10.1186/s13033-019-0272-0)
Supplement: Supplementary file 2 — Additional file 2. Interview guide. [file 13033_2019_272_MOESM2_ESM.docx]

# Additional File 2: Interview guide on health systems constraints based on the SURE framework

| Health system constraints | Accessibility of care | How accessible is this health care facility to the people to the community? Probe for distance to the clinic, social factors |
| --- | --- | --- |
|  | Financial resources | What Financial resources would be needed to implement mental health integration into primary health care? |
|  | Human resources | What human resource is available to handle the patients who come to the clinic?  How many directly assess patients? |
|  | Educational system | What do you think about the educational system for health workers in terms of mental health integration into primary health care? |
|  | Clinical supervision | How much clinical supervision do you receive in mental health integration into primary health care? |
|  | Internal communication | What is the internal communication like between the different levels of the health system or amongst the health care providers? |
|  | External communication | What is the external communication like between health workers and recipients of care? |
|  | Allocation of authority | What do you think about levels or individuals that have the authority to make decisions? |
|  | Accountability | Are authorities that make decisions accountable for the decisions they make? |
|  | Management and or leadership | What do you think about the training and leadership of your managers? |
|  | Information systems | What do you have to say about the information systems to assess and monitor needs, resource use, and utilisation of services? |
|  | Facilities | What would you comment about the supply and distribution of medical supplies and equipment to facilities?  How about maintenance of these facilities? |
|  | Patient flow processes | What processes do you have for outreach and receiving, or referring and transferring patients with mental health conditions? |
|  | Procurement and distribution systems | What is your experience with the procurement and distribution systems of drugs and other supplies in order to provide mental health services? |
|  | Incentives | What incentives do you have as a health worker to promote the mental health services? |
|  | Bureaucracy | Is there any form of bureaucracy you encounter in your practice as a health worker? |
